# The Role of Top-Down Modulation in Shaping Sensory Processing Across Brain States: Implications for Consciousness

**DOI:** 10.3389/fnsys.2019.00031

**Published:** 2019-07-24

**Authors:** Tom Sikkens, Conrado A. Bosman, Umberto Olcese

**Affiliations:** ^1^Cognitive and Systems Neuroscience Group, Swammerdam Institute for Life Sciences, University of Amsterdam, Amsterdam, Netherlands; ^2^Research Priority Area Brain and Cognition, University of Amsterdam, Amsterdam, Netherlands

**Keywords:** brain states, sensory processing, top-down modulation, feedback projections, consciousness, mismatch negativity

## Abstract

Top-down, feedback projections account for a large portion of all connections between neurons in the thalamocortical system, yet their precise role remains the subject of much discussion. A large number of studies has focused on investigating how sensory information is transformed across hierarchically-distributed processing stages in a feedforward fashion, and computational models have shown that purely feedforward artificial neural networks can even outperform humans in pattern classification tasks. What is then the functional role of feedback connections? Several key roles have been identified, ranging from attentional modulation to, crucially, conscious perception. Specifically, most of the major theories on consciousness postulate that feedback connections would play an essential role in enabling sensory information to be consciously perceived. Consequently, it follows that their efficacy in modulating target regions should drastically decrease in nonconscious brain states [non-rapid eye movement (REM) sleep, anesthesia] compared to conscious ones (wakefulness), and also in instances when a given sensory stimulus is not perceived compared to when it is. Until recently, however, this prediction could only be tested with correlative experiments, due to the lack of techniques to selectively manipulate and measure the activity of feedback pathways. In this article, we will review the most recent literature on the functions of feedback connections across brain states and based on the presence or absence of perception. We will focus on experiments studying mismatch negativity, a phenomenon which has been hypothesized to rely on top-down modulation but which persists during nonconscious states. While feedback modulation is generally dampened in nonconscious states and enhanced when perception occurs, there are clear deviations from this rule. As we will discuss, this may pose a challenge to most theories of consciousness, and possibly require a change in how the level of consciousness in supposedly nonconscious states is assessed.

## Introduction

Our brain is capable of sustaining all the functions necessary for life, from the most basic ones (breathing, autonomic regulation) to the most complex ones (language, social behavior). One function which stands out for being at the same time extremely complex to grasp, yet seamless in its presence, is consciousness. Even defining consciousness is extremely difficult, and has been a long-lasting subject of debate for philosophers and cognitive scientists (Dennett, 1991, 2018; Chalmers, 1995; Crick and Koch, 2003; Tononi and Koch, 2015; Dehaene et al., 2017; Storm et al., 2017; Lamme, 2018). In spite of this, in the last two decades significant progress has been made in the scientific study of consciousness, and in particular in the search of the neural correlates of consciousness (NCC, see Table 1 for a list of abbreviations): the neural signature of brain processes underlying consciousness (Aru et al., 2012; Koch et al., 2016). For scientists to be able to measure the NCC, however, an operational definition of consciousness is necessary.

**Table 1:** List of abbreviations.

Abbreviation	Definition
Cg1	Anterior Cingulate Cortex
DD	Deviance Detection
ECoG	Electrocorticogram
EEG	Electroencephalography
ERP	Evoked Response Potential
fMRI	Functional Magnetic Resonance Imaging
GABA	γ-Aminobutyric Acid
GNW	Global Neural Workspace
IIT	Integrated Information Theory
L1–6	Cortical layers 1–6
LFP	Local Field Potential
MCS	Minimally-conscious state
MMN	Mismatch Negativity
MMNr	Mismatch Negativity response
N1	Negative peak in visual ERPs occurring 100 ms after stimulus onset and typically associated to the MMNr
NCC	Neural Correlates of Consciousness
P300	Typical ERP elicited within a decision-making process and during oddball paradigms, with a peak occurring about 300 ms after stimulus onset
P3b	Subcomponent of the P300 ERP which has been linked to aware processing
PC	Predictive Coding
REM	Rapid Eye Movement Sleep
SSA	Stimulus-Specific Adaptation
V1-V4	Visual cortical areas 1, 4
VS	Vegetative State

Chalmers (1995) famously proposed an ontology of cognitive phenomena associated with consciousness—the easy problems of consciousness: mechanisms controlling wakefulness and sleep, the ability to report mental states, the control of behavior, etc. These phenomena—termed easy because experiments to study them may be technically challenging but pose no conceptual difficulty—are inextricably associated with conscious processing, but are not consciousness. What really defines consciousness is the subjective experience that is inextricably associated with all the cognitive phenomena mentioned above. This has been defined by Chalmers as the hard problem of consciousness because of the so-called explanatory gap (Levine, 1983) between neuron-level mechanisms and subjective experience.

The ontology first proposed by Chalmers has inspired the development of theories of consciousness aimed at addressing the nature of conscious experience, such as those proposed by Crick and Koch (1990, 2003) and Tononi and Edelman (1998a,b), and, more recently, the Integrated Information Theory (IIT; Tononi, 2004; Tononi et al., 2016). Nevertheless, Chalmer’s proposal remains highly controversial and is firmly rejected by some philosophers, chief among them Daniel Dennett (Dennett, 1991, 2018; Cohen and Dennett, 2011). According to Dennett, there is no hard problem of consciousness, but rather consciousness can be understood by studying the functions associated with it. Dennett’s approach can be seen as a theoretical foundation for another of the most influential theories of consciousness, the Global Neural Workspace (GNW) theory (Sergent and Dehaene, 2004; Baars, 2005; Dehaene et al., 2017). GNW is a theory of conscious access (Lamme, 2018; Naccache, 2018). What the theory aims to explain are the neuronal mechanisms which allow the brain to access (and subsequently report) information (Baars, 2002). This ultimately corresponds to an attempt to understand consciousness *via* addressing one of Chalmer’s easy problems, which in Dennett’s framework is all that is needed to uncover the mystery of consciousness.

The debate between these (and other) philosophical frameworks to explain consciousness, and between the different theoretical models to explain why the brain—and possibly artificial systems—is conscious is lively and at times heated, as attested by the vast recent literature on the topic (Boly et al., 2017; Odegaard et al., 2017; Dennett, 2018; Lamme, 2018; Naccache, 2018; Olcese, 2018; Olcese et al., 2018b). Nevertheless, in spite of the fundamental differences between the various frameworks to study consciousness, most theories aimed at explaining the neural mechanisms of consciousness agree on a key ingredient which makes our brain conscious: feedback connectivity. Feedforward processing consists in a progressive processing and transmission of sensory information from sensory organs up to higher-order cortical and motor regions (Lamme and Roelfsema, 2000; Lamme, 2018). While very complex forms of processing can be achieved by purely feedforward networks, as exemplified by the performance of deep artificial neural networks in the field of computer vision (LeCun et al., 2015), this is commonly agreed to occur non-consciously (Lamme, 2018). For example, both IIT and GNW concur that feedforward processing is *per se* not conscious. In IIT this can even be quantified, and purely feedforward networks (such as deep artificial neural networks) achieve a Φ value of 0 (Oizumi et al., 2014), where Φ quantifies the “level” of consciousness in IIT. Recurrent processing is, conversely, seen as a pre-requisite for consciousness by most if not all modern theories of consciousness. In IIT, as previously said, only systems with some level of integration (i.e., recursive connections) possess Φ > 0, and therefore can be considered conscious (Oizumi et al., 2014). In GNW, the feedback flow of information from frontal to posterior brain regions gives rise to the so-called global ignition which is seen as essential for consciousness (Sergent and Dehaene, 2004; Baars, 2005). Similarly, other theories, such as the recurrent processing hypothesis (Lamme, 2006, 2018), and the Predictive Coding (PC) framework (Pennartz, 2015) indicate feedback processing as a key ingredient for consciousness.

The neuroscientific evidence underlying the link between recurrent processing and consciousness has been until recently primarily limited to correlational studies done at the mesoscopic level in human subjects, *via* techniques such as electroencephalography (EEG) and functional magnetic resonance imaging (fMRI; van Gaal et al., 2011; Fahrenfort et al., 2012). These techniques, albeit powerful, lack cell-level resolution. Thus, while experiments done using EEG and fMRI can provide evidence about a generalized increase or decrease in patterns of neural activity which are compatible with modulation in feedback/recurrent coupling, they cannot discriminate what individual neurons and neuronal populations do. As an example, EEG experiments have shown that, during brain states characterized by the loss of consciousness [Non-rapid eye movement (REM) sleep, anesthesia, coma], cortical effective connectivity drops markedly (Massimini et al., 2005, 2012; Ferrarelli et al., 2010; Casarotto et al., 2016). This was thought to reflect a generalized drop in the communication between cortical areas. Recently, however, studies performed at cellular resolution in rats showed that specific forms of long-range connectivity are preserved or even enhanced in Non-REM sleep compared to wakefulness (Olcese et al., 2016, 2018a). Therefore, human studies can only give limited insight into the role of recurrent connections in conscious processing, as only average, area-level dynamics can be assessed.

Our aim is to provide an overview of the existing evidence on the role of feedback processing as a key constituent of the NCC, as provided by studies with neuron-level resolution performed in animal models. In particular, we will focus on one of the easy problems of consciousness, as defined by Chalmers (1995): the difference between wakefulness and sleep/anesthesia. We will specifically address how top-down, intra-cortical feedback varies between states of consciousness (wakefulness vs. sleep and anesthesia) and thus investigate whether the presence or absence of this form of neural dynamics can be considered a valid NCC. This will allow us to dwell into the vast literature on cortical processing in animal models, and to focus on the neocortex, i.e., the brain region which is thought to be crucial for consciousness (Koch et al., 2016). By primarily centering on differences between conscious and non-conscious brain states, we will be able to address to what extent feedback communication decreases when consciousness fades. In the first section of this manuscript, we will discuss the circuit-level mechanisms of feedforward and feedback cortical communication, and what has been reported in terms of variation across behavioral states. Next, we will zoom in on studies investigating the genesis of mismatch responses. This strongly preserved phenomenon has been shown to occur during both conscious and non-conscious states and is composed of two different components: stimulus-specific adaptation (SSA) and deviance detection (DD; Näätänen et al., 2007; Garrido et al., 2009b; Hamm and Yuste, 2016; Harms et al., 2016). These two components are, respectively, classically thought to represent feedforward and feedback forms of processing. The analysis of how these components vary across behavioral state will allow us to assess if and how feedback processing is disrupted during loss of consciousness.

## The Role of Feedback Processing Across Different Brain States

Brain state transitions can be described as the result of dynamic changes in functional long-range neuronal networks across time. Local and global connectivity greatly differ in their dynamics during the establishment of different behavioral states. While local connectivity is less affected by brain state transitions (Townsend et al., 2015; Olcese et al., 2016, 2018b; Siclari and Tononi, 2017), behavioral states exert great influences over the modulation of long-range connections (Destexhe et al., 1999; Massimini et al., 2005). In particular, a reduction in long-range connectivity has been associated with the loss in consciousness occurring in NREM sleep and anesthesia (Olcese et al., 2016; Storm et al., 2017). During wakefulness, local activity elicited within cortical microcircuits is usually broadcasted towards different brain areas through large-scale networks interconnected through feedforward and feedback projections, enabling sustained activity. This recurrent activity—necessarily supported by feedback, reentrant connections—is acknowledged to be at the basis of the NCC by several theories of consciousness (Koch et al., 2016; Lamme, 2018). In this section, we review the contribution of large-scale feedback networks in the emergence of conscious brain states, and how these networks influence the local activity of cortical microcircuits across different states.

In contrast with the predominant role recurrent connectivity plays in many of the aforementioned theories of consciousness, classical theories about sensory processing have mostly relied on the role of feedforward projections to explain how sensory stimulation can be processed (Hubel and Wiesel, 1977). Under this framework, simple stimulus features are encoded by neurons located in early sensory areas. Subsequently, this information is transferred *via* feedforward projections towards hierarchically higher areas. In these higher areas, the cumulative processing of incoming stimuli can be finally integrated into one coherent perception (Riesenhuber and Poggio, 1999). However, several cognitive processes, such as sensory perception, attention and goal-oriented behavior, have been shown to require feedback modulation—see e.g., Gazzaley and Nobre (2012), Gilbert and Li (2013), Manita et al. (2015) and Kwon et al. (2016). Furthermore, a pure feedforward approach falls short to explain how percepts—processed in a hierarchical feedforward fashion—can be accessible to conscious experience (Lamme and Roelfsema, 2000). This has been extensively investigated at the mesoscopic scale in humans (Fahrenfort et al., 2007, 2008, 2012), but the underlying circuit-level dynamics are less well understood. In a recent study investigating the neuronal responses in V1, V4, and prefrontal cortex of awake macaques during near-threshold stimulation, strong stimuli—those invariably leading to a conscious report—elicited a strong neuronal response progressing through all recorded areas, in a way compatible with the notion of inter-areal feedforward propagation (van Vugt et al., 2018). However, in case of weaker, near-threshold stimuli, signal propagation across multiple areas was highly influenced by the global pre-stimulus brain state of the animal. In detail, a computational modeling approach was used by the authors of the study to show that recurrent connectivity between hierarchically-organized areas and feedback connectivity towards sensory cortices are necessary to be able to predict neuronal responses occurring in early processing stages when stimulus detection is reported (van Vugt et al., 2018). These findings strongly suggest that feedforward activity *per se*—albeit important for perceptual processing—is not sufficient to elicit a conscious experience of a perceived stimulus. Conversely, recurrent connectivity seems to be required to support conscious responses—see also Boly et al. (2011). Nevertheless, the precise contribution of this feedback connectivity to the establishment of these networks and the mechanisms underlying local cortical circuitry modulation remains to be elucidated.

### Anatomical Organization of Recurrent Neuronal Networks

To understand what precise feedback connectivity mechanisms are contributing to consciousness, it is important to revisit some anatomical principles that govern brain connectivity in general. Different anatomical studies have estimated that a vast majority of cortical connections are essentially local (Markov et al., 2014a), organized in evolutionarily conserved microcircuits (Bosman and Aboitiz, 2015). This local architecture represents a basic organizational unit for cortical computations (Douglas and Martin, 2004; Womelsdorf et al., 2014) and, as we discuss below, the way distinct local circuits communicate with each other is tightly linked to the emergence of different brain states.

Cortical microcircuits follow a columnar organization spread across all cortical layers. The prototypical microcircuit in sensory cortices features a central granular layer (L4), receiving projections from the thalamus and other cortical layers—but see Constantinople and Bruno (2013). Supragranular layers 2 and 3 (L2/3) receive presynaptic inputs from L4, while layer 1 (L1) displays cortico-cortical fibers connecting neighborhood columns. Infragranular layers include layers 5 and 6 (L5 and L6). Neurons in these layers receive local inputs from collateral projections of L2/3 neurons and provide feedback and feedforward connectivity to different thalamic structures (Hubel and Wiesel, 1977; Shepherd, 2011; Harris and Shepherd, 2015). Both feedforward and feedback projections have a strong preference for targeting areas that are close to their origin (Markov et al., 2014b). Supporting this highly redundant local connectivity, an estimated amount of 20% of the total connectivity is organized through long-range interareal connections (Markov et al., 2014a). These projections follow a well-defined pattern of connectivity (Felleman and Van Essen, 1991; Markov et al., 2014a,b). Feedforward projections preferentially originate in supragranular layers and target granular layers. Conversely, feedback connections arise from infragranular layers and avoid targeting granular layers (Felleman and Van Essen, 1991).

**Figure 1:**
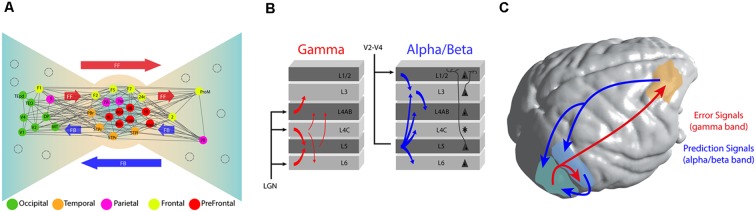
Functional architecture of cortical connectivity. **(A)** Brain topology as a Bow-Tie organization. This brain organization arises from the connectivity profile obtained from retrograde tracer studies. It defines a heavily interconnected core of fronto-parietal areas and the ties based in feedforward (FF) and feedback (FB) connections from the core. As expected, early sensory areas are located more at the extremes of the tie. Adapted with permission from Markov et al. (2013b). **(B)** Representation of the directed oscillatory influences (measured by Granger causality) over the cortical column in area V1 of the monkey. Gamma (30–90 Hz) oscillations are predominantly feedforward and they target granular and supragranular layers. Alpha/beta oscillations (roughly 8–30 Hz, combined) are predominantly feedback. They originate at hierarchically higher areas and target predominantly infragranular layers. Adapted with permission from van Kerkoerle et al. (2014). **(C)** Long and short-range connections implementing a Predictive Coding (PC) framework of sensory processing. Under such framework, a predictive model is originated at prefrontal cortex and local areas and channeled towards early sensory areas by long and short-range connections respectively, using low frequency (alpha and beta) oscillations. Error signals, originated from sensory areas are broadcasted towards hierarchically higher brain areas using high-frequency oscillations.

The quantification of this pattern of connectivity (Ercsey-Ravasz et al., 2013; Markov et al., 2014b) has suggested an important structural heterogeneity across brain areas, arranged in what Kennedy and colleagues have denominated a Bow-Tie organization (Markov et al., 2013b). Under such an organization, a subset of association areas including frontal, parietal, and temporal cortices appear to be heavily interconnected *via* recurrent connections, forming a central core of brain areas (Figure 1A). This highly interconnected core is characterized by a high prevalence of long-distance connections departing from this core to other brain regions (Markov et al., 2013a). At the periphery, two different sets of regions are linked to the core through feedback and feedforward connections: a first group composed of primary and secondary sensory regions, and a second group consisting of premotor areas. These two groups are interconnected from the first to the second group *via* direct feedforward connections, and from the second to the first one *via* direct feedback connections (Ercsey-Ravasz et al., 2013).

This organizational principle encompasses several advantages. It reduces brain wiring and volumetric surface, yet increases the efficiency of information transfer and computational speed (Markov et al., 2013b). It reveals the importance of a fronto-parietal network, fundamentally characterized by a high prevalence of long-range connections, which some authors have suggested to be compatible with a GNW organization (Ercsey-Ravasz et al., 2013; Markov et al., 2013a), and provides an evolutionary advantage for long-range communication and cortical coordination of brain dynamics (Bosman and Aboitiz, 2015).

### Functional Dynamics of Feedback and Feedforward Connections: A Possible Role of Brain Oscillations

A bow-tie brain topology strongly relies on the functionality of long-range feedback connections to support sensory processing. As stated before, classical (and mainly feedforward) theories about perception are insufficient to explain sensory processing under such organizational principles. Conversely, the PC framework offers important insights into sensory perception in a view that is compatible with the notion of neuronal entrainment. PC is rooted in the tradition of inferential models of brain perception (Friston, 2010; Clark, 2013). Under this formulation, feedback projections transmit to hierarchically lower areas a generative model of sensory perception. In turn, feedforward projections transfer a signal error from the model, derived from the comparison between the existing model and the incoming sensory signals (Rao and Ballard, 1999; Bastos et al., 2012). Accordingly, recurrent (i.e., local) interactions between models and error signals at the level of cortical microcircuits are thought to improve the generation of statistical inferences about sensory perception. It has been argued that cortical microcircuit architectures can effectively implement PC computations. Under such scenario, local cortical microcircuits effectively integrate data and models originated from long-range feedforward and feedback networks, respectively (Bastos et al., 2012, 2015; Fontolan et al., 2014; Chao et al., 2018). Nevertheless, it is important to note that a PC framework can infer statistical regularities, but it cannot specify why data from different types of sensors would be consciously experienced (Pennartz, 2009). In other words, a PC framework helps to explain the importance of feedback connectivity but does not offer a solution to the hard problem of consciousness.

Experimentally, the study of neuronal dynamics underlying cortical computations across areas requires the utilization of techniques able to record several areas and spatial levels simultaneously. From a myriad of emerging techniques (Adesnik and Naka, 2018), ensemble recordings and surface local field potentials (LFPs), recorded—respectively—by high-density laminar probes and electrocorticograms (ECoGs), are considered a primary choice due to their capacity to simultaneously record multiple local neuronal assemblies through cortical layers (Lewis et al., 2015; Pesaran et al., 2018). Laminar recordings in early visual areas have shown that LFP oscillations—a prominent feature of field recordings which has been associated with several perceptual and cognitive functions (Bosman et al., 2014; Fries, 2015)—are compartmentalized across cortical layers (Figure 1B). High-frequency oscillations (e.g., in the gamma frequency band, between 30–90 Hz) are observed mostly in supragranular layers. Conversely, lower frequency bands, such as alpha (8–12 Hz) and beta (13–30 Hz), are observed mostly in infragranular layers (von Stein et al., 2000; Buffalo et al., 2011; van Kerkoerle et al., 2014).

Brain rhythm compartmentalization appears to have a functional role during inter-areal communication (Fries, 2015). Recent studies enabling simultaneous recordings across multiple brain areas have demonstrated that directed long-range interactions can be exerted across different frequency band channels, a scenario compatible with the PC framework (Bastos et al., 2012, 2015; Fontolan et al., 2014; Michalareas et al., 2016; Chao et al., 2018). A study using high-density laminar profile in V1 of awake monkeys has shown that, while gamma band responses initiate in L4 and propagate through supra and infragranular layers of higher visual areas, alpha oscillations travel in the opposite direction (van Kerkoerle et al., 2014, Figure 1B). These relationships between frequencies and cortical layers are consistently observed through the cortical hierarchy. Using ECoGs in awake monkeys, Bastos et al. (2015) showed that, while feedforward directed influences (measured in terms of Granger causality) are observed through gamma oscillations across eight cortical regions recorded simultaneously, feedback influences across the visual hierarchy are consistently carried out by beta oscillations, and a similar functional connectivity pattern has been observed in humans (Michalareas et al., 2016). Furthermore, new studies have shown that feedback modulation can be updated continuously using error signals broadcasted from sensory areas to prefrontal cortex (Chao et al., 2018; Figure 1C). Neurons located in prefrontal cortex have the ability to integrate such error signals and continuously submit updated versions of the model through feedback projections to temporal areas (Chao et al., 2018). In early sensory cortices, the efficiency of feedforward frequency coupling—measured as an increment of gamma frequency band coherence between V1 and V4—increases after augmented top-down beta frequency band modulation (Richter et al., 2017). Importantly, optimized feedforward efficiency improves conscious access of perceived stimuli (Lamme and Roelfsema, 2000; Boly et al., 2011), and behavioral responses during attentional tasks (Rohenkohl et al., 2018), indicating that the extent of long-range feedback modulation correlates with conscious accessibility and enhanced behavioral performance.

Thus, simultaneous recordings across multiple areas using LFP rhythmic fluctuations have consistently shown that long-range feedback signals, possibly originating from highly interconnected hubs comprising anterior regions of the brain, effectively modulate the activity of early sensory regions. This feedback modulation ultimately facilitates feedforward communication, behavioral performance and conscious reporting of perceived stimuli. A PC architecture, implemented across local-to-global anatomical connections and dynamical LFP oscillatory phase relationships, may support this organization (Olcese et al., 2018b).

However, while several studies have shown the importance of PC architectures and their oscillatory dynamics during stimulus processing, the exact contribution of these processes during different brain states still needs to be elucidated (Olcese et al., 2018b). The observation of local and global network dynamics across wide-brain areas across different behavioral states is a crucial step to understand what the mechanisms underlying these behavioral and network transitions are. Feedforward gamma connectivity globally decreases during deep sleep states, as compared to awake states, in intracranial recordings in epilepsy patients (Mikulan et al., 2018), but there are no consistent reports about low-frequency (and putatively feedback) phase relationships during sleep, in spite of the reduced role of feedback during non-conscious states (Boly et al., 2011). During anesthesia, a pharmacologically-induced brain state, quantitative EEG studies have shown that sedation with propofol is accompanied by a decreased posterior alpha and increased frontal/central beta power (Gugino et al., 2001; Akeju and Brown, 2017). This shift has been extensively studied and is thought to emerge from a disruption of prefrontal circuits created by a strong low-frequency thalamocortical synchronization (Vijayan et al., 2013; Flores et al., 2017). Yet, the detailed effects of anesthesia on cortico-cortical synchronization remain elusive. Finally, a promising line of research about the effects of feedback connectivity in patients with consciousness disorders has been developed in recent years. Feedback influences, but not feedforward ones, seem to be compromised in these patients (Boly et al., 2011), in agreement with the notion that feedback connections are important to sustain conscious activity. Intriguingly, a temporary recovery in the consciousness state of a subgroup of patients has been observed following the administration of the GABA modulator Zolpidem (Hall et al., 2010; Williams et al., 2013). Zolpidem administration reduces EEG power and coherence at 6–10 Hz frequencies (Williams et al., 2013). Surprisingly, however, Zolpidem seems to elevate beta frequency power at anterior regions following its administration. These effects seem to be mediated by thalamocortical interactions but is not clear whether direct cortico-cortical communication is also affected (Hall et al., 2010; Williams et al., 2013). Future studies might help to unveil the exact nature of these effects.

In conclusion, long-range feedback connections are an essential component of conscious brain states and determine how sensory information is processed at the local level (*via* both feedforward and recurrent mechanisms). Yet, the underlying circuit-level mechanisms remain poorly understood. Several studies have outlined the organizational principles—both anatomical and functional—of feedback connections in the context of sensory processing. However, contrasting results have been presented on how feedforward and feedback functional connectivity vary as a function of the state of consciousness. Oscillatory dynamics during sensory processing may provide a framework to understand the role of feedback projections, but these phenomena involve a wide range of brain circuits, operating both at feedforward and feedback level and both at local and global scales. Consequently, one possible solution to understand the role of feedback projections and the underlying circuit mechanisms in conscious processing is to focus on processes that are purely feedback in nature, and which are thought to be exclusively dependent on long-range, top-down mechanisms.

## Mismatch Negativity: Preserved Top-Down Modulation During Non-conscious Brain States?

The mismatch negativity response (MMNr) is a well-studied electrophysiological phenomenon that occurs in the human brain after violation of a rule, which is established by a sequence of repeated stimuli (Näätänen et al., 2007; Garrido et al., 2009b). The MMNr reflects the brain’s capacity to automatically detect unpredicted sensory changes in our environment, without the need for attention (Tiitinen et al., 1994; Näätänen et al., 2001; Stefanics et al., 2014), and is impaired in several psychological afflictions such as schizophrenia, attention-deficit hyperactive disorder and psychosis (Erickson et al., 2016; Näätänen et al., 2016). In recent years the interest for the MMNr has grown considerably, especially in relation to the hierarchical PC framework, bringing a new plethora of insights from both human and rodent experiments.

**Figure 2:**
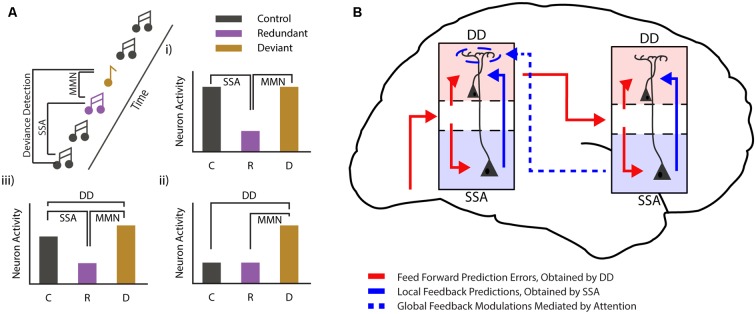
Mismatch negativity during different states of consciousness. **(A)** Cartoon showing the commonly used auditory oddball paradigm and the different comparisons that can be used to differentiate between stimulus-specific adaptation (SSA) and deviance detection (DD). Three bar graphs (i—iii) show the hypothetical cases in which classical MMN van be observed. In one case (i) MMN can be fully explained by SSA. Another example (ii) shows a pure deviance detecting neuron, while the final example (iii) shows MMN as it is most likely found in awake subjects, with both SSA and DD. **(B)** Schematic showing how local DD may be maintained during loss of consciousness, while global predictions are lost. Global, long-range feedback projections are reduced during loss of consciousness, while local connectivity is maintained. This suggests that low-level predictions might arise from local connectivity (for example from deep to superficial layers), while more complex, global prediction requires top-down modulation. These top-down projections mostly target interneurons in the most superficial layer of the cortex, where they are in a prime position to modulate neurons from both the superficial and the deep layers of the cortex.

The MMNr is often studied using an oddball paradigm, in which a particular stimulus (standard) is repeatedly presented to a subject, but sometimes unpredictably changed to a different, unexpected stimulus (deviant, see Figure 2A). The idea behind this paradigm is that the repetition of a particular stimulus or event leads to the formation of a prediction related to the frequency or probability at which this event occurs. Stimuli that deviate from the frequently presented one elicit a strong mismatch response. Thus, the MMNr can be interpreted as a prediction error to a stimulus that does not match the statistical regularities of sensory stimuli being perceived, thus updating our internal representation of the world (Friston, 2005; Garrido et al., 2009b; Stefanics et al., 2014).

### Different Components of the Mismatch Negativity Response

The MMNr can be split into two functionally distinct components (Hamm and Yuste, 2016; Harms et al., 2016). The first component arises as an effect of stimulus repetition. Repetition of a particular event leads to a decreased neuronal response to that event, a phenomenon often referred to as repetition suppression or SSA. SSA has been found in many different neural systems and at various levels: from single neurons in the cortex of primates (Miller and Desimone, 1994), cats (Ulanovsky et al., 2003) and rodents (Taaseh et al., 2011), to field and imaging recordings in rodents (Chen et al., 2015; Hamm and Yuste, 2016; Parras et al., 2017; Hamm et al., 2018) and humans (Garrido et al., 2009a). While SSA closely resembles the MMNr originally described in humans (Ulanovsky et al., 2003; Nelken and Ulanovsky, 2007) it does not inherently have the second crucial component of the MMNr, true deviance detection (DD). For DD to occur the increased response to the deviant compared to the standard stimulus should not be fully explained by the decrease in neuronal firing caused by SSA (i.e., it should not simply be equal to the response elicited when the standard stimulus is not presented in a sequence). Rather, the increase in this response should have added value in detecting the occurrence of a deviant or irregular event (Hamm and Yuste, 2016; Harms et al., 2016). In this paragraph, we will discuss the relationship between SSA and DD, and how they are related to the MMNr.

Repetition of a particular stimulus leads to neuronal adaptation in the response to this stimulus, which can be characterized by the decrease of the neuronal response over repetitions. There are several hypotheses on which neuronal mechanisms could underlie this adaptation. Some models suggest that relatively simple feedforward mechanisms, such as neuronal fatigue, may fully explain the effects of stimulus repetition (Grill-Spector et al., 2006). Indeed, it is possible that SSA can be explained, at least in part, by purely feedforward mechanisms (Garrido et al., 2009a; Farley et al., 2010; May and Tiitinen, 2010), where (synapses of) neurons that are responsive to the standard stimulus adapt over time, resulting in responsive depression, while the neurons responding to the deviant stimulus are “fresh” and are activated in full. A more likely case, however, is that multiple mechanisms contribute to repetition suppression under different conditions (Grill-Spector et al., 2006).

Another possible view on SSA comes from the PC framework. According to this framework, the decrease in neuronal activation upon repeated stimulation reflects the brain’s ability to predict that stimulus (Friston, 2005, 2008, 2010). This is achieved by neuronal processes optimized to probabilistically represent causes of sensory inputs. These processes can be seen as the building of an internal model representing our external environment. Keeping this model up-to-date requires a constant interaction between top-down predictions and bottom-up prediction errors (Bastos et al., 2012). An increased ability of this generative model to predict sensory inputs is reflected in a decrease of neuronal activity necessary to update the model. In other words, SSA can be considered as a product of perceptual learning where the predicted part of a sensory input is “explained away.”

DD, on the other hand, is an increased neuronal response to deviant stimuli, and can be considered a neural correlate of error signaling or memory update, where the prediction does not match the actual input and an adjustment of the generative model is called for.

Though SSA has been extensively studied in animal models, until recently it remained unclear whether MMN and more specifically DD could also be found in rodents (Taaseh et al., 2011; Chen et al., 2015; Hamm and Yuste, 2016; Parras et al., 2017; Hamm et al., 2018). These studies, paving the way for investigating the microcircuit-level mechanisms of DD, not only found that true DD is present in the rodent neocortex, but that SSA and DD can be observed within different time-windows of the whole MMNr (Chen et al., 2015; Hamm and Yuste, 2016). While SSA is mainly found in earlier time components (40–80 ms after stimulus onset), DD is predominantly visible in a later time window (120–240 ms after stimulus onset). Furthermore, these separate components possibly involve distinct cortical networks and neuronal populations (Chen et al., 2015; Natan et al., 2015; Hamm and Yuste, 2016; Parras et al., 2017; Hamm et al., 2018). Though questions remain on how experimental results regarding SSA and DD in animal models compare to MMNr in human subjects, it can be assumed that they are all part of the same PC process.

### MMN and the Canonical Microcircuits for Predictive Coding

The generation of the MMNr is a hierarchical process. While forms of SSA can already be found in the early stages of sensory processing such as the thalamus (Natan et al., 2015; Parras et al., 2017; Hamm et al., 2018), DD seems to be absent in these early processing steps and appears later in the hierarchy, starting from (primary) cortical areas. Moreover, the proportion of neurons showing true DD increases when going up in the cortical hierarchy (Parras et al., 2017; Hamm et al., 2018). This has been taken as an indication that, while SSA can arise from purely feedforward processing streams, DD is more likely dependent on recurrent or feedback processing. This notion fits very well with the connectivity scheme proposed by the PC theory (Bastos et al., 2012; Auksztulewicz and Friston, 2016)—see “The Role of Feedback Processing Across Different Brain States” section.

In the PC framework, top-down predictions play an important role in eliciting mismatch or error-signals. Excitatory connections from (higher-order) cortical areas send predictions to predominantly inhibitory neuronal population in earlier sensory areas (Bastos et al., 2012). These inhibitory neurons enable local pyramidal cells to compare sensory inputs with these predictions, resulting in processes such as SSA (Bastos et al., 2012; Auksztulewicz and Friston, 2016; Yarden and Nelken, 2017). Indeed, it has been shown that deactivating auditory cortex reduces SSA in the superior colliculus of rats, though SSA is not completely extinguished (Anderson and Malmierca, 2013). Furthermore, inhibiting projections from the anterior cingulate cortex (Cg1)—a high-order cortical area involved in modulating visual responses (Zhang et al., 2014; Fiser et al., 2016)—to the primary visual cortex (V1) abolishes DD at a population level in V1 itself (Hamm et al., 2018). This would support a primary role for long-range feedback projections—which are also thought to be essential for conscious processing—in enabling DD. It is important to note, however, that, when splitting neuronal activity into populations that, are “adapting,” “deviance detecting” or “non-modulated,” the activity of “deviance detecting” neurons in V1 remains intact (Hamm et al., 2018). In other words, while DD seems to disappear when looking at the gross activity of V1, it is preserved at the single-neuron level. This raises the question of how important long-range feedback connections are for facilitating mismatch responses in early sensory cortices, and what their exact role is. Is it possible for example that mismatches caused by low-complexity stimulus features such as visual orientation can be solved within the visual cortex through local (recurrent) connectivity?

**Figure 3:**
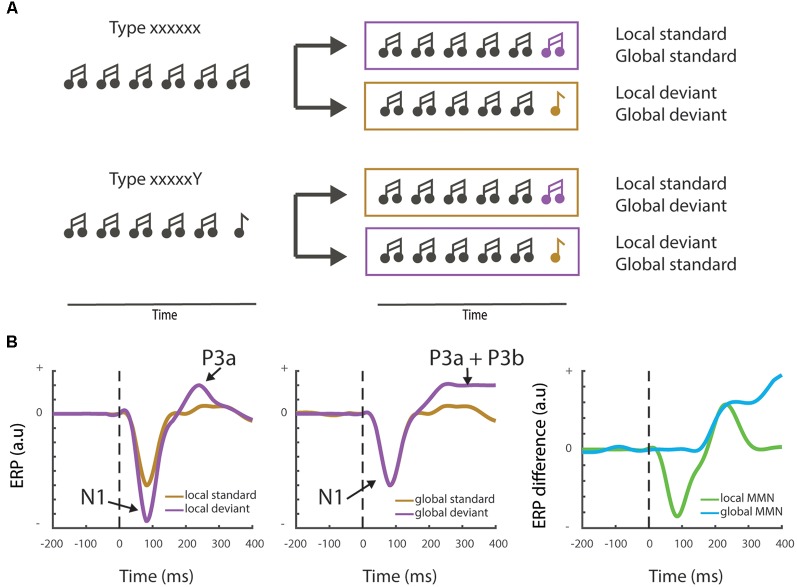
Local vs. global mismatch responses. **(A)** Cartoon showing the local-global oddball paradigm and the distinct stimulus trains that differentiate between local and global deviants. Note that in the “xxxxxY” block the change of the final “Y” to an “x” stimulus induces a global deviant (“xxxxxx”), whereas it remains a standard stimulus at the local level. **(B)** Schematic examples (not actual data—see e.g., Bekinschtein et al., 2009 or Strauss et al., 2015 for measured electrophysiological traces) showing the typically observed field responses during a local-global mismatch paradigm. The left panel shows the classical Mismatch negativity response (MMNr) where the N1 component is more negative for the deviant response compared to the standard response. The local MMNr also generally show an early P3a response. Global deviants (middle panel) may not elicit a change in the N1 response, but induce a maintained sensory novelty or P3b response. The right panel depicts the difference waves (deviant—standard) generally used to visualize the MMNr.

To answer this question, we should make a distinction between local/low-level expectations that can operate independently of active cognitive processing (the pre-attentive part of the MMNr), and global/higher-order generalizations that require cognitive or attentional modulation. One way to make this dissociation is by using an adapted version of the oddball paradigm. The local-global paradigm dissociates between two types of predictions, based on local probabilities vs. global rules (Figure 3A). It uses blocks of stimuli where a sequence of for instance five stimuli (“xxxxx”) is presented interspersed with infrequent blocks in which the final stimulus is changed to a rare/deviant stimulus (“xxxxY”; Bekinschtein et al., 2009; Wacongne et al., 2011). Importantly, in other blocks, the frequent sequence is of the “xxxxY” type, while the deviant sequence consists of five identical stimuli (“xxxxx”). Evoked response potential (ERP) recordings reveal that the fifth, locally deviant tone of the standard “xxxxY” type, although fully predictable, still elicits a MMNr. However, only the rare violation sequence (or global deviant), which contains the five identical tones “xxxxx,” elicits a distinct and later novelty response, the “late positive complex” or P3b wave (Bekinschtein et al., 2009, Figure 3B). This P3b component has been associated with consciousness through processes such as attention and awareness—see Chennu and Bekinschtein (2012) for an overview. In the next paragraph, we will discuss how the MMNr and related ERP components, such as the P3b wave, are influenced by different states of awareness and how these changes are supported by (long-range) feedback connections.

### Mismatch Negativity in Relation to Consciousness

The MMNr has been investigated extensively in relation to different states of consciousness. It has been of interest in cognitive research because of its robustness: MMN can be observed in different states of consciousness, be it awake, asleep or anesthetized (Atienza et al., 2002; Koelsch et al., 2006). This has led to the belief that MMN is an automated response not dependent on active, conscious processing (Näätänen et al., 2001; Stefanics et al., 2014). However, ERP components related to the MMNr, especially the P3b wave, can be altered by conscious processes such as attention and awareness (Woldorff et al., 1998; Näätänen et al., 2007). Consequently, it has been hypothesized that, as the MMNr may be a key NCC, changes in its properties can be used to track different states of awareness, such as sedation or loss of consciousness. In a clinical setting, the estimation of a patient’s level of consciousness may be of paramount importance for the correct diagnosis of disorders of consciousness.

In cognitive electrophysiology, the P3b wave is a part of the most widely studied P300 ERP component. It has been proposed as a marker of conscious perception of salient events or stimuli (Sutton et al., 1965) and more specifically as a sign of the “top-down” deployment of selective attention to task-relevant stimuli. While the P3b response, similarly to the MMNr, is often studied using a form of the oddball paradigm, the processes underlying the two responses may be very different. For example, the processing of statistical irregularities that elicits the MMNr is distinct from, and may not necessarily result in, conscious awareness of a stimulus (Chennu and Bekinschtein, 2012). Furthermore, the MMNr has been reported in humans over various states of consciousness (Atienza et al., 2002; Koelsch et al., 2006; Bekinschtein et al., 2009), whereas the P3b component seems dependent on aware processing of the stimulus (Sergent et al., 2005) and is diminished in states of impaired consciousness (Boly et al., 2011; Strauss et al., 2015; Nourski et al., 2018). Nevertheless, although the P3b response is usually considered an event separate from the MMNr, the two are often temporally overlapping. For example, recent studies in mice assess oddball and mismatch responses in temporal windows up to 400 ms after the onset of sensory stimuli (Chen et al., 2015; Hamm and Yuste, 2016), beyond the typical N1 response which peaks 100 ms after stimulus onsets and is associated with the MMNr in human EEG studies. Here, we will consider the P3b as an ERP component related to the MMNr. Future experiment will need to assess whether the P3b can be considered as a long-latency DD component of the MMNr or rather a separate neuronal event.

Upon loss of consciousness, detection of local deviants remains, at least partially, present in the human brain (Strauss et al., 2015; Nourski et al., 2018). Detection of global deviants is, on the other hand, absent during states of unconsciousness and deep sleep (Strauss et al., 2015; Nourski et al., 2018). Thus, global MMNr and the related P3b component are dependent on cognitive processes that are unavailable during loss of consciousness, whereas local DD remains partially present. While both the local DD component of the MMNr and the P3b response have been hypothesized to be dependent on temporal/frontal feedback connections (Friston, 2005, 2010; Garrido et al., 2009b; Buschman and Kastner, 2015), these differences indicate that distinct forms of feedback projections may be involved in the detection of local vs. global deviants.

As mentioned before (local) MMN has been reported under different states of awareness in both human and animal experiments (Atienza et al., 2002; Koelsch et al., 2006; Parras et al., 2017). However, questions have been raised on whether these reports truly reflect the whole MMN process, including its deviance detecting component, or whether MMN under low conscious conditions can be fully explained by SSA. In a recent study, Strauss et al. (2015) showed disruption of the local MMNr at intermediate time components during non-REM sleep. These mid-range components have previously been associated with the error-signaling or deviance detecting processes underlying the MMNr (Wacongne et al., 2012). These results indicate that the feedback modulation that is necessary to support not only global but also local DD might be missing during non-conscious brain states. Indeed, another study showed that patients in a vegetative state (VS) display reductions in feedback connectivity, while feedforward connections are maintained (Boly et al., 2011)—but see King et al. (2011). Moreover, intermediate (DD-related) components of the MMNr are not present in these patients, whereas they are still reported in patients in a minimally conscious state (MCS). Thus, the main difference between MCS and VS patients seems to be the reduction of feedback connectivity in VS patients, leading to a disruption in DD. Finally, a recent animal study that supports the previous findings showed that inactivation of feedback connections from frontal cortex to visual cortex disrupts local DD processes in the latter (Hamm et al., 2018).

Altogether, the studies mentioned in the previous paragraphs indicate that: (1) feedback connections are necessary to maintain the (local) DD component of MMN, and (2) feedback connectivity is strongly reduced during non-conscious brain states. Interestingly, however, the same study showed that, while DD disappears upon inhibiting feedback projections when looking at the population level (i.e., in terms of multi-unit activity), at the single-neuron level DD is unchanged (Hamm et al., 2018). Moreover, other studies have reported both MMNr and (local) DD at the single-neuron level in anesthetized animals (Taaseh et al., 2011; Parras et al., 2017). Thus, while DD seems to disappear in population-level recordings during non-conscious states or when prefrontal cortex is inhibited (Boly et al., 2011; Strauss et al., 2015; Hamm et al., 2018), it is preserved in the cortex when looking at the single-neuron level (Parras et al., 2017; Hamm et al., 2018). These discrepancies highlight some of the knowledge gaps in our current understanding of the role of feedback projections in MMN generation and, potentially, their involvement in conscious responses.

Several distinct mechanisms may explain the aforementioned discrepancies. First, local DD might primarily depend—as classically hypothesized and similarly to global DD—on long-range feedback projections. During anesthesia/loss of consciousness such feedback is reduced (but not completely eliminated), and this dampens the extent of local DD in such a way that it is no longer visible at the population level. A second possibility is that, at least for local mismatches, DD is primarily due to feedforward or local (recurrent) mechanisms (Figure 2B). Feedback projections, in a manner similar to top-down attention, amplify and synchronize DD across neurons. This makes DD visible at the population level and allows the “error” signal to be more effectively transmitted to higher-order areas. Current experimental evidence does not allow us to disambiguate between these two scenarios. In fact, no experiment has yet been able to either monitor or abolish (or even identify) all sources of feedback that might play a role in the generation of the local DD response. Consequently, only hypotheses can be made on its genesis. Addressing this problem is essential not only to provide an understanding of the mechanisms underlying MMN (which is clinically relevant for the diagnosis of several neuropsychiatric disorders), but also to better understand the neural basis of consciousness.

## Discussion: Are Feedback Connections Essential for Consciousness?

Long-range feedback projections have been hypothesized to play an essential role in the generation of consciousness, yet conclusive evidence for this is lacking. First (“The Role of Feedback Processing Across Different Brain States” section), while it has been shown that long-range feedback is dampened in non-conscious brain states (Boly et al., 2011), some studies found exceptions to this general trend (Chennu et al., 2014), even for cortico-cortical top-down projections. Other studies even showed that, during Non-REM sleep, some forms of inter-areal communication may even be enhanced compared to wakefulness (Olcese et al., 2018a). One possible solution to this discrepancy between theoretical frameworks and experimental evidence may be that not all long-range, cortico-cortical feedback projections are equally important in the generation of consciousness. Identifying which feedback pathways support conscious processing is crucial to develop better markers for the level of consciousness. State-of-the-art diagnostic tools for linking brain dynamics to the level of consciousness, such as the perturbational complexity index (Casali et al., 2013), are able to characterize inter-areal communication in general but lack the specificity to probe specific connections. Second (“Mismatch Negativity: Preserved Top-Down Modulation During Non-conscious Brain States?” section), the role of feedback is being questioned even for fundamental brain mechanisms which have long been held to depend on it and to be hallmarks of conscious states, such as the DD component of the MMNr. Not only may the DD component still be present in non-conscious states, but also it may not be fully dependent on feedback projections (Hamm et al., 2018). In particular, a key question is whether feedback projections contribute to the generation of the DD response, or only facilitate it *via* synchronizing neuronal activity. In other words, is the DD response an error signal computed in higher-order areas (as the PC framework suggests), or is it generated locally and top-down modulation only amplifies it? And, if the latter proves to be the case, what are the local/recurrent mechanisms underlying DD, and is this consciously perceived? Finally, it remains to be addressed whether the feedback functional dynamics responsible for the DD component of the MMNr are merely a correlate of consciousness (NCC) or rather represent a key mechanism underlying the emergence of consciousness. Addressing these questions is crucial for us to understand the neural bases of consciousness, yet remains challenging and requires an interdisciplinary approach.

First, an experimental paradigm in which DD responses are preserved in non-conscious states at the single-neuron—but not population—level needs to be developed for both humans and animals. Correlational human experiments involving intra-cranial recordings are needed to investigate whether single-neuron DD is consciously perceived even in the absence of long-range feedback and population-level signals. This can be done *via* retrospective reports (Koch et al., 2016; Siclari et al., 2017), and will allow us to understand if feedback projections are involved in generating conscious percepts, or rather enable to report those. The answer to this question is critical to reveal which cortical regions are involved in the genesis of conscious perception (Koch et al., 2016; Boly et al., 2017; Lamme, 2018). Second, causal explanations provided by animal experiments are subsequently crucial to go beyond the correlational level and reveal what the exact role of feedback projections is in the generation of DD responses. By leveraging and integrating techniques such as high-channel count electrophysiology, two-photon calcium imaging and optogenetics, it will be possible to uncover whether (and which) feedback projections play a role in the computation of the DD response, or whether they only have a modulatory nature. One additional intriguing outcome of such experiments (which can be only achieved by combining human and animal experiments) would also be to finally reveal the differential role of top-down projection from frontal to posterior cortices, or within the posterior cortex (parietal, temporal and occipital regions), and address the debate on which cortical regions support conscious processing (Koch et al., 2016; Boly et al., 2017; Odegaard et al., 2017; Lamme, 2018).

## Conclusions

How does the brain transform sensory stimuli into perceptual experiences? And how does it generate an internal model of the world? These are questions that have puzzled philosophers since the dawn of civilization. Neuroscientists have in the past decades started to formulate theories on how the brain might generate consciousness, yet all theories are lacking in terms of specific, circuit-level mechanisms. Theories focused on addressing the hard problem of consciousness (e.g., IIT) provide a foundational framework of the properties a conscious system needs to possess, but do not specify how this is reflected in terms of neuron-level (micro)circuits. The same is true for neurophysiology-based theories (recurrent processing, GNW, PC framework). General, mesoscopic-scale principles have been outlined, yet very limited insight is provided about the corresponding single-neuron correlates. Irrespective of one’s philosophical or theoretical stance (see the debate between Chalmers and Dennett), this is a key question which was until recently not addressable, but that developments in neurotechnology make within reach.

While functional long-range feedback projections are generally agreed to be closely associated to the presence of consciousness, recent studies have begun to indicate that not all feedback (not even within the cortex) may play the same role. The precise function of feedback has proven to be elusive even for phenomena (such as DD) that have historically been considered dependent on feedback. In order to eventually understand consciousness, therefore, two questions must be addressed: which forms of feedback support consciousness—and are not just a correlate of it? And how do they operate, at the microcircuit level?

Answering these questions will make it possible to eventually test the mechanistic role of feedback connections in generating consciousness. Is proper feedback sufficient to generate consciousness, or only necessary, provided that other conditions are met? If proper feedback were sufficient to generate consciousness in information processing systems, any biological or artificial device would have the potential for being conscious (although so far only brains may have achieved the right form of feedback). Although certain configurations of feedback projections are most likely essential to achieve functions normally associated with consciousness—Chalmer’s easy problems, but see also Dehaene et al. (2017)—we concur with Tononi and Koch (2015) that merely reproducing a function does not imply possessing consciousness (Tononi and Koch, 2015; Tononi et al., 2016). Thus, while a computer simulation with a proper, yet virtual, form of feedback would likely be able to match conscious systems from a functional point of view, we surmise that a proper physical substrate is a pre-requisite to achieve real consciousness, i.e., experience—see Tononi and Koch (2015) for an in-depth discussion on the topic. Rather, we support the hypothesis that proper feedback projections are necessary to achieve consciousness in computational systems (neuron-based, artificial, etc.), but only if these possess an appropriate physical substrate (Tononi and Koch, 2015; Tononi et al., 2016). Nevertheless, these fascinating questions can only be achieved after a deeper understanding of the role of feedback projections in conscious processing. This will finally allow, we believe, to unveil the mystery which the mechanisms of consciousness still represent.

## Author Contributions

TS, CB and UO wrote the manuscript.

## Conflict of Interest Statement

The authors declare that the research was conducted in the absence of any commercial or financial relationships that could be construed as a potential conflict of interest.
